# Plantar Mechanical Stimulation Maintains Slow Myosin Expression in Disused Rat Soleus Muscle via NO-Dependent Signaling

**DOI:** 10.3390/ijms22031372

**Published:** 2021-01-29

**Authors:** Kristina A. Sharlo, Inna I. Paramonova, Irina D. Lvova, Ekaterina P. Mochalova, Vitaliy E. Kalashnikov, Natalia A. Vilchinskaya, Sergey A. Tyganov, Tatyana S. Konstantinova, Tatiana F. Shevchenko, Grigoriy R. Kalamkarov, Boris S. Shenkman

**Affiliations:** 1 Myology Laboratory, Institute of Biomedical Problems RAS, 123007 Moscow, Russia; sharlokris@gmail.com (K.A.S.); irrrra1@yandex.ru (I.D.L.); mochalova_ekaterina@lenta.ru (E.P.M.); xenoling@yandex.ru (V.E.K.); vilchinskayanatalia@gmail.com (N.A.V.); sentackle@yandex.ru (S.A.T.); bshenkman@mail.ru (B.S.S.); 2 Emanuel Institute of Biochemical Physics, RAS, 123007 Moscow, Russia; ts.konst@gmail.com (T.S.K.); tatianashevchenko@rambler.ru (T.F.S.); kalam2@rambler.ru (G.R.K.)

**Keywords:** hindlimb unloading, L-NAME, MyHC, plantar mechanical stimulation, soleus muscle

## Abstract

It was observed that gravitational unloading during space missions and simulated microgravity in ground-based studies leads to both transformation of slow-twitch muscle fibers into fast-twitch fibers and to the elimination of support afferentation, leading to the “switching-off” of postural muscle motor units electrical activity. In recent years, plantar mechanical stimulation (PMS) has been found to maintain the neuromuscular activity of the hindlimb muscles. Nitric oxide (NO) was shown to be one of the mediators of muscle fiber activity, which can also promote slow-type myosin expression. We hypothesized that applying PMS during rat hindlimb unloading would lead to NO production upregulation and prevention of the unloading-induced slow-to-fast fiber-type shift in rat soleus muscles. To test this hypothesis, Wistar rats were hindlimb suspended and subjected to daily PMS, and one group of PMS-subjected animals was also treated with nitric oxide synthase inhibitor (L-NAME). We discovered that PMS led to sustained NO level in soleus muscles of the suspended animals, and NOS inhibitor administration blocked this effect, as well as the positive effects of PMS on myosin I and IIa mRNA transcription and slow-to-fast fiber-type ratio during rat hindlimb unloading. The results of the study indicate that NOS activity is necessary for the PMS-mediated prevention of slow-to-fast fiber-type shift and myosin I and IIa mRNA transcription decreases during rat hindlimb unloading.

## 1. Introduction

It is known that gravitational unloading during a space flight or under simulated microgravity in ground-based studies, immobilization, bed rest, spinal isolation results in a slow-to-fast shift by altering the expression of slow myosin heavy chain gene (*MyHC*) in skeletal muscle [1,2,3,4,5,6]. The mechanisms of this shift remain largely unexplored. Nevertheless, it is known that the key factor regulating the type of a muscle fiber in adult animals is the mode of contractile activity of this fiber [7]. The maintenance of normal neuromuscular activity of the hindlimb muscles depends on the gravitational forces of the Earth detected by specific cutaneous sensory receptors in the sole that transmit afferent signals to the central nervous system, providing neuromotor activity of the hindlimb muscles [8].

Ground-based models of microgravity (rat hindlimb suspension, “dry immersion”) lead to an almost complete disappearance of the neuromuscular activity of antigravity soleus muscle according to electromyography data [9,10,11]. Observations show that the elimination of support afferentation is the main mechanism leading to the “switching-off” of the electrical activity of the slow motor units of postural muscles during gravitational unloading [12,13,14,15,16]. It has been demonstrated that increasing sensory input by plantar mechanical foot stimulation (PMS) during space flights [17] and in ground-based experiments including human volunteers [18,19] and rats [13,20] leads to an increase in the neuromuscular activity of hindlimb muscles. Mechanical stimulation of the support zones of the feet can be an effective measure to prevent the negative effects of gravitational unloading and various pathological conditions. PMS has been found to maintain contractile properties, cytoskeleton integrity, intrinsic stiffness and fiber size in the human soleus during the 7-day “dry immersion” [14,15,21,22]. In the described experiments, the partial prevention of the slow fiber transformation into fast ones during mechanical stimulation of the human subjects feet support zones was also shown. However, the signaling mechanisms of these effects have remained unexplored until recently.

One of the key signaling pathways regulating the expression of the slow myosin isoform is the calcineurin/NFATc1 signaling pathway [23]. In active muscle fiber, calcineurin, activated by calmodulin-bound calcium ions, dephosphorylates NFATc1 and promotes its translocation into the myonuclei. In the nuclei, NFATc1 directly interacts with MEF2 transcription factors that specifically bind the promoter of the slow *MyHC* gene and activate its expression [24,25]. Thus, intense transcription of the “slow” *MyHC* gene is triggered.

Under muscle unloading, the NFATc1 content in the nuclei and the MHC slow isoform mRNA transcription decreases [26,27]. In our previous work, we have demonstrated that mechanical plantar foot stimulation of rat sole exerts a direct effect on the calcineurin/NFATc1 signaling pathway and partially prevents the decrease in type I MHC expression as well as myosin phenotype shift towards the fast direction under gravitational unloading within 3 days of exposure [26].

Another mechanism for regulating the expression of slow myosin heavy chain isoforms is associated with AMP- and Ca-dependent phosphorylation of histone deacetylase-4 (HDAC4) in the active muscle, which prevents its translocation into the nuclei, where HDAC4 blocks the expression of the slow myosin gene [28]. Earlier in our laboratory, it was shown that the accumulation of HDAC4 in myonuclei under gravitational unloading occurs due to dephosphorylation of AMP-activated protein kinase (AMPK) and it is accompanied by a decrease in the expression of the *myh7* gene [29].

One of the key signaling substances which reflects the level of activity of skeletal muscle is nitric oxide. Numerous experiments have shown a decrease in the content of neuronal NO synthase after hindlimb suspension of rodents [30,31], head-down tilt bedrest and human immersion [21,32,33], as well as a decrease in neuronal NO synthase mRNA expression after 14 days of hindlimb suspension [30,34].

In skeletal muscle, nitric oxide can regulate NFATc1 content in muscle nuclei by inactivating GSK-3beta kinase, which phosphorylates NFAT and leads to its removal from the nucleus [27,35]. Nitric oxide can also lead to inactivation of class IIa histone deacetylases in skeletal muscle [36] and to activation of AMPK and CaMK II that remove HDAC4 from the muscle nuclei [36,37,38,39].

In the article by Sharlo et al., 2019 we suggested a hypothesis that nitric oxide is a key factor preventing the transformation of muscle fibers during PMS, but this assumption has not yet been verified [26]. In this study we suggest that one of the most important consequences of the increased activity of the postural muscle during the stimulation of support afferents under gravitational unloading is the maintenance of the physiological level of nitric oxide production. If this assumption is true, the effect of preventing slow-to-fast transformation of the soleus muscle fibers during plantar mechanical foot stimulation of an animal under gravitational unloading will be neutralized by the action of the nitric oxide synthase inhibitor L-NAME.

Based on this hypothesis, the aim of the work is to study the role of nitric oxide and NO-dependent signaling pathways in maintaining the stability of the expression of slow myosin during plantar mechanical stimulation under gravitational unloading.

## 2. Results

### 2.1. NO Production

We measured NO content in soleus muscle by an electron paramagnetic resonance method. After 7 days of hindlimb unloading, the NO content was significantly decreased in the 7HS group compared to the control group (*p* < 0.05). PMS application during the hindlimb unloading prevented NO decrease in the 7HS + PMS group so that NO content in this group did not differ from the control group. L-NAME administration during PMS resulted in complete prevention of the PMS effects on NO content so that NO content in the 7HS + LN + PMS group was equal to the 7HS group (Table 1).

**Figure 1:**
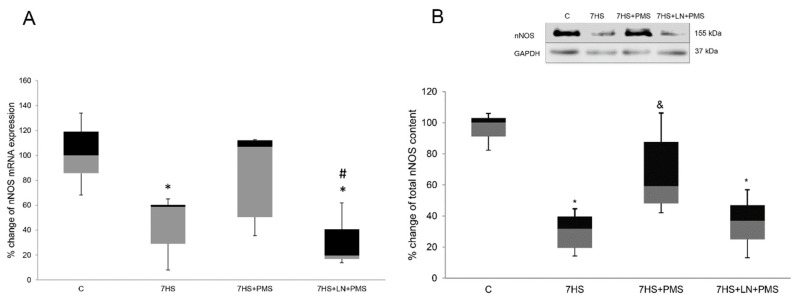
PCR-analysis of nNOS mRNA expression level (**A**), Western-blot analysis of total nNOS content (**B**) in rat soleus muscle in control group (C), 7-day hindlimb-suspended group (7HS), 7-day hindlimb suspended group with plantar mechanical stimulation (7HS + PMS), 7-day hindlimb suspended group with plantar mechanical stimulation+ L-NAME introduction (7HS + LN + PMS). Data are shown as % of the control group. *—significant difference from the control group. &—significant difference from 7HS group. #—significant difference from 7HS + PMS group (*p* < 0.05). Box plots show 25–75 percentiles and median values and the whiskers represent the minimum and the maximum; *n* = 8/group.

We also measured nNOS mRNA expression and total nNOS content in soleus muscle tissues of experimental animals. Both nNOS mRNA expression and protein content decreased after 7 days of hindlimb unloading compared to C group. In 7HS + PMS group there were no significant differences from control in both nNOS mRNA and protein, and protein content of nNOS was significantly increased in this group compared to 7HS group (Figure 1A,B). In 7HS + LN + PMS group, nNOS protein content and mRNA expression was equal to 7HS group.

### 2.2. Myosin mRNA Transcription and Fiber-Type

**Figure 2:**
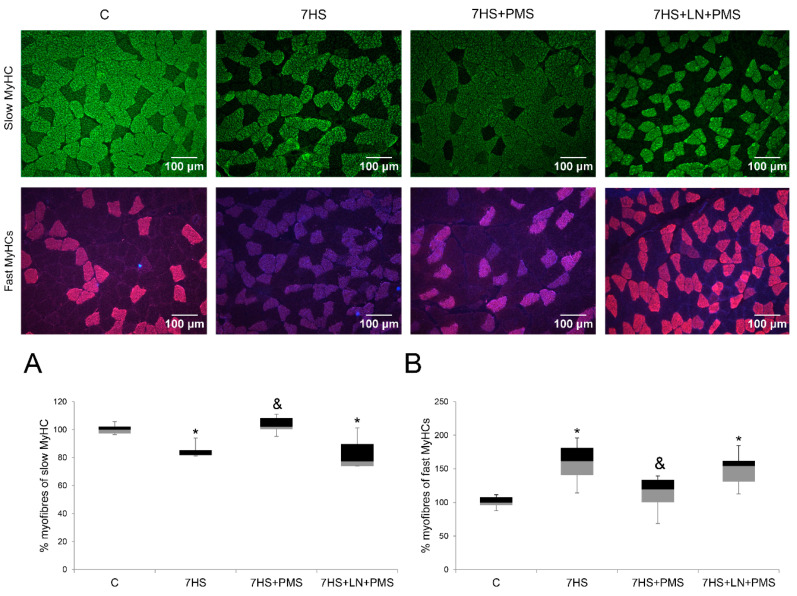
Immunohistochemical analysis of slow (**A**) and fast (**B**) fiber number on rat soleus cross-sections in the control group (C), 7-days hindlimb-suspended group (7HS), 7-days hindlimb suspended group with plantar mechanical stimulation (7HS + PMS) and 7-day hindlimb suspended group with plantar mechanical stimulation+ L-NAME introduction (7HS + LN + PMS). Data are shown as % of the control group. *—significant difference from the control group. &—significant difference from 7HS group (*p* < 0.05). Box plots show 25–75 percentiles and median values and the whiskers represent the minimum and the maximum; *n* = 8/group.

The immunohistochemical evaluation of the slow-to-fast fiber-type ratio showed that the percentage of slow-type fibers significantly reduced and the percentage of fast-type fibers significantly increased in the 7HS group compared to the control group, while the slow-to-fast fiber-type ratio in the 7HS + PMS group was equal to the control one. L-NAME administration blocked the effect of PMS so that in the 7HS + LN + PMS group the slow-to-fast fiber-type ratio was equal to the 7HS group value (Figure 2A,B). 

**Figure 3:**
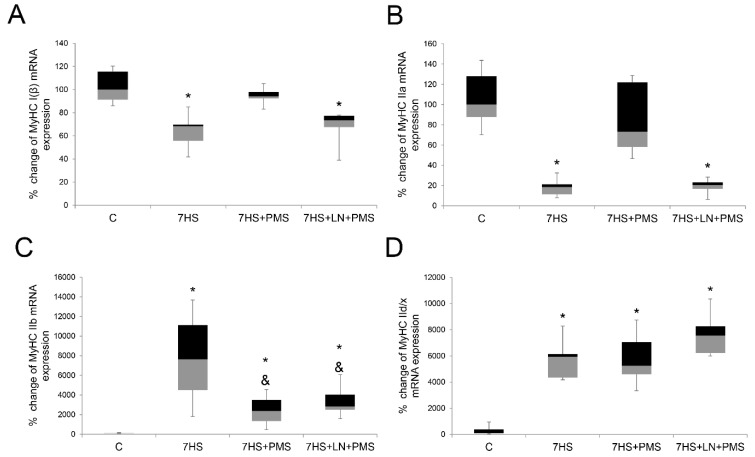
Expression levels of *MyHC I(β)* (**A**), *MyHC IIa* (**B**), *MyHC IIb* (**C**), *MyHC IId/x* (**D**) mRNAs in rat soleus muscle in the control group (C), 7-day hindlimb-suspended group (7HS), 7-day hindlimb suspended group with plantar mechanical stimulation (7HS + PMS), 7-day hindlimb suspended group with plantar mechanical stimulation+ L-NAME introduction (7HS + LN + PMS). Data are shown as % of the control group. *—significant difference from the control group. &—significant difference from 7HS group (*p* < 0.05). Box plots show 25–75 percentiles and median values and the whiskers represent the minimum and the maximum; *n* = 8/group.

Seven days of rat hindlimb unloading were followed by a significant reduction of type I and IIa myosin mRNA transcription in the 7HS group compared to the C group, and plantar mechanical stimulation prevented the declines so that in the 7HS + PMS group both type I and type II a myosin RNA transcription levels did not differ from the control and they were both significantly higher than in the 7HS group. At the same time, in the 7HS + LN + PMS group both type I and type IIa myosin RNAs significantly declined compared to the C group and were equal to the 7HS group, so we suggest that L-NAME blocks the protective effect of PMS on slow and II a myosin mRNAs transcription (Figure 3A,B). Fast-type myosin IIB and IId/x mRNAs both significantly increased in the 7HS group compared to the control group. PMS led to a significant downregulation of II B myosin mRNA in the 7HS + PMS group compared to the 7HS group, although II B mRNA content in this group was still higher than in the control group. L-NAME administration did not interfere with this effect of PMS so that in the 7HS + LN + PMS and in the 7HS + PMS groups the levels of myosin IIB mRNA were equal. At the same time, IId/x myosin mRNA transcription did not differ among 7HS, 7HS + PMS and 7HS + LN + PMS groups as well (Figure 3C,D).

### 2.3. Micro-RNA-Dependent Signaling

**Figure 4:**
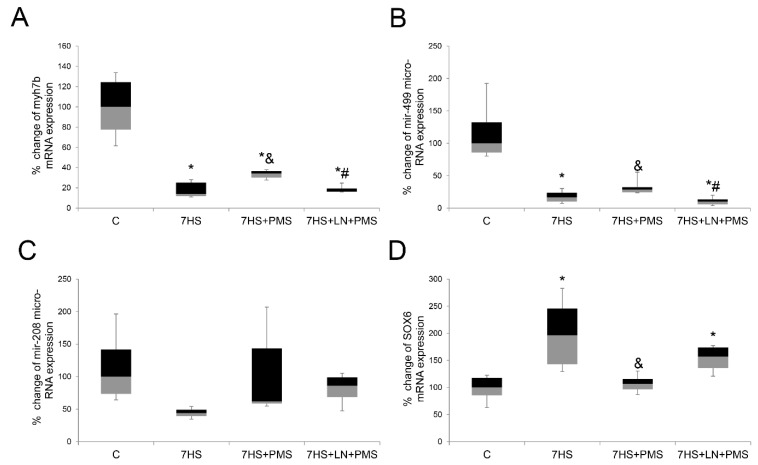
Expression levels of myh7b mRNA (**A**), mir-499 micro-RNA (**B**), mir-208 micro-RNA (**C**) and SOX6 mRNA (**D**) in rat soleus muscle in control group (C), 7-day hindlimb-suspended group (7HS), 7-day hindlimb suspended group with plantar mechanical stimulation (7HS + PMS), 7-day hindlimb suspended group with plantar mechanical stimulation+ L-NAME introduction (7HS + LN + PMS). Data are shown as % of the control group. *—significant difference from the control group. &—significant difference from 7HS group. #—significant difference from 7HS + PMS group (*p* < 0.05). Box plots show 25–75 percentiles and median values and the whiskers represent the minimum and the maximum; *n* = 8/group.

The level of pre- mir-499 mRNA of slow-tonic myosin *myh7b* and the level of mir-499 micro-RNA significantly reduced in the 7HS and the 7HS + LN + PMS groups compared to the C group. In the 7HS + PMS group the levels of both *myh7b* mRNA and mir-499 were significantly higher than in the 7HS and the 7HS + LN + PMS groups, although they were still lower than in the C group (Figure 4A,B). 

Surprisingly, the level of mir-208, which is a product of type I myosin exon, did not significantly differ among the experimental groups, although it had a decline trend in the 7HS group compared to the control one (*p* = 0.1). The level of SOX6 slow-type gene repressor mRNA, the transcription of which is directly inhibited by mir-499 and mir-208b, was twofold higher in the 7HS group compared to the control group, while in the 7HS + PMS group its level was equal to the control group. In the 7HS + LN + PMS group, the level of SOX6 mRNA was also significantly higher than in the C group (Figure 4C,D). 

### 2.4. Calcineurin/NFAT Signaling

**Figure 5:**
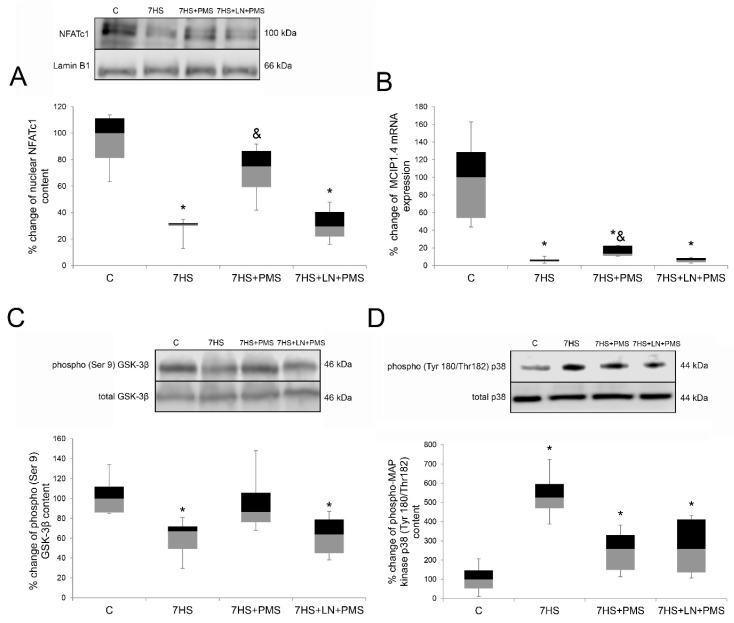
Western-blot analysis of NFATc1 nuclear content (**A**), phospho- GSK-3β(Ser 9) content (**C**), phospho-p38(Tyr 180/Thr182) content (**D**) and expression level of MCIP1.4 mRNA (**B**) in rat soleus muscle in control group (C), 7-day hindlimb-suspended group (7HS), 7-day hindlimb suspended group with plantar mechanical stimulation (7HS + PMS), 7-day hindlimb suspended group with plantar mechanical stimulation+ L-NAME introduction (7HS + LN + PMS). Data are shown as % of the control group. *—significant difference from the control group. &—significant difference from 7HS group (*p* < 0.05). Box plots show 25–75 percentiles and median values and the whiskers represent the minimum and the maximum; *n* = 8/group.

After 7 days of hindlimb unloading the nuclear content of NFATc1 transcription factor and the level of NFATc1-dependent MCIP1.4, mRNA transcription were both significantly reduced in the 7HS group compared to the C group (*p* < 0.05). PMS application during hindlimb unloading partially prevented NFATc1 nuclear content decline and MCIP1.4 mRNA downregulation in the 7HS + PMS group so that both of these parameters were significantly higher compared to the 7HS group. NFATc1 nuclear content in the 7HS + PMS group was equal to the control. However, MCIP1.4 RNA in the 7HS + PMS group did not reach the control level and was still decreased compared to the control one. L-NAME administration during PMS resulted in complete prevention of the PMS effects on NFATc1 nuclear content and MCIP1.4 mRNA transcription, so that both of these parameters in the 7HS + LN + PMS group were similar to the 7HS group (Figure 5A,B).

NFATc1 can be phosphorylated and in this way inactivated by two major protein kinases, GSK-3beta and MAP kinase p38, so we measured the level of phosphorylation of these kinases. 

The level of GSK3beta Ser 9 phosphorylation significantly fell down after 7 days of unloading, indicating the increased activity of GSK3beta in the 7HS group compared to the control group. In the 7HS + PMS group the level of GSK3beta Ser 9 phosphorylation significantly excessed the 7HS group value and was the same as the C group, while in the 7HS + LN + PMS group the level of GSK3beta Ser 9 phosphorylation was equal to that in the 7HS group (Figure 5C). The level of MAP kinase p38 Tyr 180/Thr182 phosphorylation raised in all the hindlimb-unloaded groups compared to the C group (*p* < 0.05). In the 7HS group, MAP kinase p38 Tyr 180/Thr182 phosphorylation level was fivefold above the control, while in 7HS + PMS and in 7HS + LN + PMS groups it increased only threefold, although there were no significant differences either between 7HS and 7HS + PMS groups or between 7HS and 7HS + LN + PMS groups (Figure 5D). 

**Figure 6:**
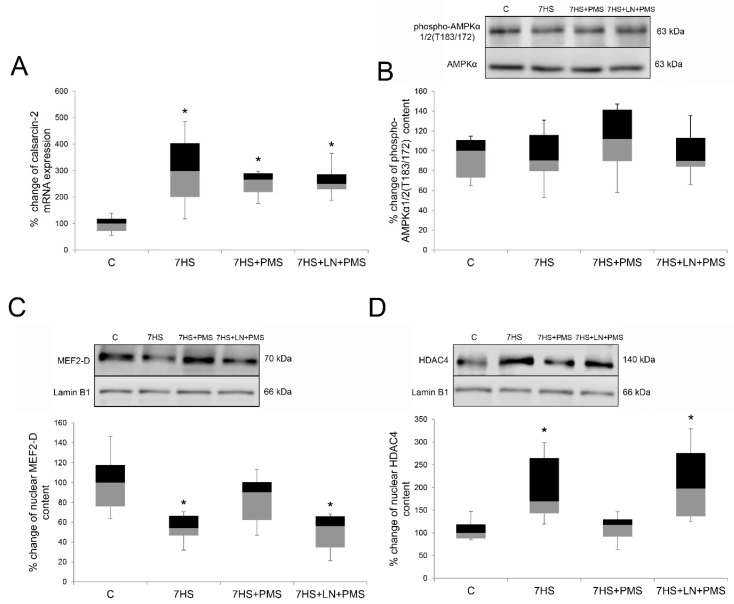
Expression level of calsarcin-2 mRNA (**A**), Western-blot analysis of phospho-AMPKα1/2(T183/172) content (**B**), Western-blot analysis of MEF2-D (**C**) and HDAC4 (**D**) nuclear content in rat soleus muscle in control group (C), 7-day hindlimb-suspended group (7HS), 7-day hindlimb suspended group with plantar mechanical stimulation (7HS + PMS), 7-day hindlimb suspended group with plantar mechanical stimulation+ L-NAME introduction (7HS + LN + PMS). Data shown as % of the control group. *—significant difference from the control group. Box plots show 25–75 percentiles and median values and the whiskers represent the minimum and the maximum; *n* = 8/group.

The mRNA content of the endogenous calcineurin inhibitor calsarcin-2 raised threefold in the 7HS group compared to the C group (*p* < 0.05). Neither PMS nor L-NAME affected this increase so that in all the hindlimb-unloaded groups calsarcin-2 mRNA transcription significantly increased as compared to the C group (Figure 6A).

### 2.5. AMPK/MEF2-D Signaling and Epigenetic Regulation

The level of AMPKα1/2 Thr 183/172 phosphorylation did not differ among all the experimental groups (Figure 6B).

After 7 days of hindlimb unloading the nuclear content of MEF2-D transcription factor significantly decreased in the 7HS group compared to the control one, and the nuclear HDAC4 content was upregulated (*p* < 0.05). PMS application during the hindlimb unloading partially prevented HDAC4 nuclear content raise and nuclear MEF2-D reduction in 7HS + PMS group so that both of these parameters did not differ from the control. HDAC4 and MEF2-D nuclear contents in the 7HS + PMS group were similar to the C group. L-NAME administration during PMS resulted in complete prevention of the PMS effects on HDAC4 and MEF2-D nuclear content so that both of these parameters in the 7HS + LN + PMS group were equal to the 7HS group (Figure 6C,D).

**Figure 7:**
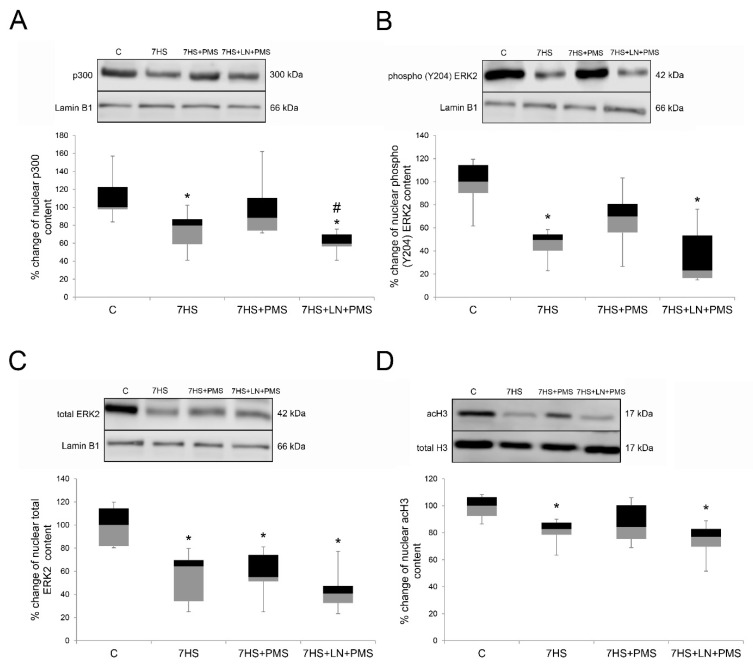
Western-blot analysis of p300 (**A**), phospho-ERK2(Y204) (**B**), total ERK2 (**C**) and acH3 (**D**) nuclear content in rat soleus muscle in control group (C), 7-day hindlimb-suspended group (7HS), 7-day hindlimb suspended group with plantar mechanical stimulation (7HS + PMS), 7-day hindlimb suspended group with plantar mechanical stimulation+ L-NAME introduction (7HS + LN + PMS). Data are shown as % of the control group. *—significant difference from the control group. #—significant difference from 7HS + PMS group (*p* < 0.05). Box plots show 25–75 percentiles and median values and the whiskers represent the minimum and the maximum; *n* = 8/group.

The nuclear content of p300 and the level of MAP kinase ERK2 (Y204) phosphorylation both significantly decreased in the 7HS group compared to the C group (*p* < 0.05). PMS application during hindlimb unloading partially prevented p300 nuclear content decrease and nuclear ERK2 (Y204) phosphorylation level decrease in 7HS + PMS group, so that both of these parameters did not differ from the control group. P300 nuclear content and nuclear ERK2 (Y204) phosphorylation level in the 7HS + PMS group were equal to the control. L-NAME administration during PMS resulted in complete prevention of the PMS effects on p300 nuclear content and nuclear ERK2 (Y204) phosphorylation level, so that both of these parameters in the 7HS + LN + PMS group were equal to the 7HS group (Figure 7A,B). The nuclear content of MAP kinase ERK2 significantly decreased in all the hindlimb-unloaded groups compared to the control group (*p* < 0.05). ERK2 nuclear content did not differ among 7HS, 7HS + PMS and 7HS + LN + PMS groups as well (Figure 7C).

Histones H3 are a substrate for both HDAC4 and p300 acetylation. After 7 days of hindlimb unloading the nuclear content of acetylated H3 was significantly reduced in the 7HS group compared to the control group (*p* < 0.05). In the 7HS + PMS group the level of acH3 was the same as in the C group, while in the 7HS + LN + PMS group the level of acH3 was equal to that in the 7HS group (Figure 7D).

## 3. Discussion and Conclusions

### 3.1. NO Production

We detected a significant decrease in the NO content in soleus muscle after 7 days of hindlimb unloading (group 7HS). These data correspond to our previous results after 7 days and 14 days of rat hindlimb unloading [27,34]. However, the literature data about the NO content in soleus muscles of unloaded animals are discordant. Some authors suggest that NO production during hindlimb unloading is upregulated, which leads to muscle atrophy [31,40,41], while others showed the positive effects of NO on the unloaded animals soleus muscles [34,42]. The authors supporting the NO increase hypothesis explain this increase by a translocation of the neuronal NO synthase from the membrane to the cytoplasm. The translocation was observed in several studies [31,41], but currently there are no published data documenting the activation of non-membrane-bound NOS. The direct measurements of NO content in soleus muscles by the EPR method were conducted both by our group and by Suzuki and colleagues, and the results obtained by the two groups are opposite one another. This contradiction may be explained by the differences in the experimental procedures. In our experiment the soleus muscles of the experimental animals were frozen in liquid nitrogen, and all the subsequent procedures, including EPR spectrum recording, were conducted at the temperature of liquid nitrogen (similar to the procedure described in [43]). At the same time, Suzuki and colleagues minced the soleus muscle tissue and measured NO content at room temperature [31] so these differences in the experimental procedures could lead to discordant results. A number of evidences concerning the states of NO-dependent signaling pathways, such as Akt-independent GSK-3beta activation during the early stage of hindlimb unloading [26,44], indirectly support the suggestion that NO content in soleus muscle tissue is more likely to decrease than increase under unloading conditions. However, neuronal NOS does not significantly contribute to prevention of atrophy during soleus muscle stretching upon rat hindlimb suspension, but is involved in the maintenance of expression of some muscle proteins, in particular in the regulation of MHC I mRNA expression upon muscle stretching [45]. Moreover, in our previous article we described that L-arginine administration to unloaded animals prevented slow myosin mRNA expression decrease but did not prevent soleus muscle atrophy [27]. 

The decrease of nNOS mRNA and total protein content is in accordance with multiple previous data about nNOS content under functional muscle unloading [21,33,45]. It was shown that mechanical loading can regulate both NO production by NO-synthase and has a long-term positive effect on nNOS expression [30]. Based on these data we can suggest that 7 days of plantar mechanical stimulation prevents NO level decrease in soleus muscles by affecting nNOS mRNA transcription. We were rather surprised to find out that this regulation is NO-dependent; however, NO is a potent epigenetic regulator, so it could be possible that nNOS is positively regulated by its own product.

The observed maintenance of NO content in the PMS-subjected group is in good agreement with the previously shown PMS-induced prevention of nNOS content downregulation after 3 days of rat hindlimb unloading and 3 days of human dry immersion [21,26]. The administration of NOS inhibitor L-NAME to the animals from another PMS-subjected unloaded group (7HS + LN + PMS) promoted the decrease in NO content in their soleus muscles, as it had been expected. We suggest that the NO maintaining in the plantar stimulated group is mediated by the PMS-induced soleus muscle neuromuscular activation. It was demonstrated that increasing sensory input by performing plantar mechanical stimulation (PMS) to the soles of the feet during microgravity in humans [17] and in ground-based experiments in both humans [19,21] and rats [13] leads to an increase in neuromuscular activation of the lower limb muscles [46]. NO production was shown to be activated by muscle electrostimulation as well as by mechanical stress, and both these conditions are tightly connected with muscle activity [30,47]. Based on these data, we suggest that NO is one of the key mediators of skeletal muscle neuromuscular activity. 

### 3.2. Myosin mRNA Transcription and Fiber-Type 

The decrease in slow-type fibers percentage and type I and II a myosins mRNA transcription and the upregulation of IIB and IId/x mRNA transcription after rat hindlimb unloading, had been shown in numerous studies [34,48,49,50,51]. However, there is much less information concerning the effect of the plantar mechanical stimulation on slow-to-fast fiber-type ratio or myosin mRNA transcription. The observed slow myosin mRNA transcription increase, the maintenance of slow-to-fast fiber-type ratio and the attenuated IIB myosin mRNA transcription raise correspond to our previous data of the 3-days rat hindlimb unloading with PMS [26]. At the same time, we had not observed the maintenance of IIa myosin mRNA transcription during PMS in the previous study, although during the current experiment we observed a strong protective effect of PMS on IIa myosin mRNA transcription. This discrepancy may be explained by the different durations of these two experiments, as PMS may require more time to affect myosin IIa mRNA transcription than type I myosin mRNA transcription. 

The observed effects of NOS inhibitor administration on myosin isoforms mRNA transcription and slow-to-fast fiber-type ratio make it evident that NOS activity is necessary for the PMS-mediated prevention of slow-to-fast fiber-type shift and myosin I and IIa mRNA transcription decrease during rat hindlimb unloading. The NO-dependent activation of slow-type myosin mRNA expression and slow-type fiber percentage increases were shown in numerous studies both in vivo and in vitro [27,35,37,38,52,53]. Therefore, based on these results we can assume that the effects of plantar mechanical stimulation on slow-to-fast fiber-type ratio are mediated by PMS-dependent NOS activation and prevention of nitric oxide content decrease in the unloaded soleus muscle.

At the same time, the effect of PMS on the transcription of IIB myosin mRNA does not depend on NOS activity and the myosin II d/x mRNA transcription does not respond to PMS. Therefore, we suggest that during the hindlimb unloading slow-type myosin and fast-type myosin mRNA transcription are controlled independently.

### 3.3. Micro-RNA-Dependent Signaling

Myosin type I mRNA in skeletal muscle fibers produces mir-208 micro-RNA, encoded in one of the gene introns. Mir-208 induces the transcription of slow-tonic myosin *myh7b* gene [54]. *myh7b* mRNA is not translated into a protein molecule, but produces micro-RNA 499 [55]. Both micro-RNAs 208 and 499 target the 3′ UTR of the transcriptional repressor SOX6, which is involved in the repression of slow fiber-type genes, in particular the slow myosin gene, and downregulates SOX6 mRNA transcription [54,56]. Based on these facts, we decided to analyze the contents of *myh7b* and SOX6 mRNAs in soleus muscles of the experimental animals. 

The observed declines in *myh7b* gene and its product mir-499 mRNA transcription, as well as SOX6 mRNA transcription increase after 7 days of HS correspond to the data of McCarthy and colleagues, who observed an MiR-499 transcription decrease at this time-point [54]. In our study, it is shown for the first time that PMS application during the hindlimb unloading facilitates the myh7b/MiR-499/SOX6 signaling pathway. These data correspond to the previously observed effect of L-arginine administration on myh7b and SOX6 expression [27]. However, the effect of PMS on MiR-499 was far more profound than the effect on MiR-208, so it is unlikely that the PMS effect on MiR-499 is mediated only by slow myosin and MiR-208 transcription upregulation. As MiR-499 was shown to be upregulated by MEF2 transcription factors, we suggest that the maintenance of MEF2-D nuclear content in the PMS group could also affect MiR-499 upregulation and subsequent SOX6 mRNA decrease [57]. It should be noted that the effect of PMS on myh7b/MiR-499/SOX6 signaling as well as on MEF2-D nuclear accumulation, is mediated by NO synthase activity in this experiment, so this pathway may be one of the mechanisms of NO-dependent slow myosin upregulation in soleus muscle. 

### 3.4. Calcineurin/NFAT Signaling

Another signaling pathway controlling slow myosin mRNA transcription is calcineurin/NFATc1. NFATc1 nuclear accumulation was shown to be regulated by nitric oxide in C2C12 myotubes [53]. The decrease in nuclear NFATc1, which is one of the key transcriptional activators of slow myosin gene, as well as the decrease in its downstream MCIP1.4 mRNA after 7-days unloading are in good agreement with our results for this time-point [27]. Plantar mechanical stimulation prevented nuclear NFATc1 decrease and to a lesser extent the decrease in MCIP1.4 mRNA transcription, and both of these effects are blocked in the NOS-inhibited group. These data correspond to our previous results of L-arginine treatment of hindlimb of unloaded animals [27]. These findings support our previous suggestion that PMS upregulates NFATc1 myonuclear content and transcriptional activity by NO-dependent signaling [26]. However, MCIP1.4 mRNA transcription restoration in the 7HS + PMS group is far less prominent than NFATc1 nuclear content restoration. This result may evidence that the unloading-induced NFATc1 nuclear content decrease is not the only cause of NFATc1-dependent transcription decrease during unloading, and some other regulators may control NFATc1 activity, as NFATc1 may undergo intranuclear inactivation, accumulating in the heterochromatin regions of the myonuclei [58]. 

NFATc1 exit from muscle nuclei was shown to be controlled by phosphorylation by GSK3beta and/or MAP kinase p38 [53,59,60]. The observed GSK3beta Ser 9 phosphorylation decrease followed by the kinase activation in the 7HS group as well as the prevention of this effect by PMS, corresponds to our previous data [26]. The PMS effect on the GSK3beta Ser 9 phosphorylation strongly depends on NOS inhibition, so we suggest that PMS may regulate GSK3beta activity by the well-studied nNOS/cGMP/PKG signaling pathway [27,35,53,61]. 

The unloading-induced MAP kinase p38 Tyr 180/Thr182 phosphorylation increase leading to the kinase activation was shown on different stages of rat hindlimb unloading, although the upstream mechanisms of this increase are still unknown [62,63]. PMS led to the partial prevention of p38 phosphorylation increase, so p38 partial inactivation may also contribute to the PMS-induced nuclear NFATc1 restoration. However, NOS inhibition does not interfere with PMS effect on p38 phosphorylation, unlike NFATc1 myonuclear content, so NFATc1 nuclear content is more likely to be regulated by GSK3beta than by p38 MAP kinase. The calsarcin-2 mRNA transcription level also seems not to contribute much to the NFATc1 myonuclear content, and calsarcin-2 mRNA transcription regulation still remains unclear, as PMS downregulated its expression after three days of rat hindlimb unloading but not after seven days of unloading [26]. However, L-arginine administration to hindlimb unloading animals led to both NO content increase and calsarcin-2 mRNA downregulation in soleus muscles of the experimental animals. This discrepancy suggests that calsarcin-2 expression regulation may be indirectly regulated by NO content as well as by some other signaling pathways.

### 3.5. Epigenetic Regulation

Nitric oxide is a well-known epigenetic regulator in various tissues which promotes histone acetylation by blocking histone deacetylases and activating histone acetyltransferases [64]. However, the detailed mechanisms of NO-dependent histone acetylation are unknown in most cases. In our study we determined the decrease of nuclear acH3 after seven days of HS. Plantar mechanical stimulation prevented the nuclear acH3 decrease and this effect was blocked in the NOS-inhibited group. 

We suggest that these NO-dependent epigenetic changes reflect the NO-dependent activation of HATs and inactivation of HDACs in soleus muscle tissues of the animals subjected to PMS, and changes of HAT/HDACs activity could also affect slow myosin mRNA transcription, as the promoter of slow myosin was shown to undergo the unloading-induced acetylation decline [65].

The decrease in nuclear content of HAT p300 after the hindlimb unloading is in agreement with earlier data [66,67]. Plantar mechanical stimulation prevented nuclear p300 decrease and prevented HDAC4 nuclear accumulation. As the effect of PMS on both HDAC and HAT nuclear contents was blocked in the NOS-inhibited group, we suggest that nitric oxide upregulates p300 nuclear content and downregulates HDAC4 nuclear content, thereby affecting intranuclear activities of these two regulators and H3 acetylation level. It should be noticed that p300 was shown to acetylate and activate the transcription factor NFATc1, so NO-dependent nuclear p300 raise could also contribute to NFAT-dependent transcription activation.

HDAC4 was shown to block the slow myosin mRNA transcription by deacetylating MEF2 transcription factors. The observed nuclear MEF2-D decrease and nuclear HDAC4 increase correspond to our previous data [29,67]. However, in the study of Paramonova et al. 2020, the nuclear content of HDAC4 increased by 36 percent unreliably after 7 days of HS. It is noteworthy that in the present study the MEF2-D myonuclear content decreases after 7 days of unloading, while nuclear HDAC4 increases after 7 days of unloading. However, the cause of MEF2-D myonuclear export is unclear now. Plantar mechanical stimulation prevented not only nuclear HDAC4 increase, but nuclear MEF2-D decrease also, and both of these effects are blocked in the NOS-inhibited group. These findings support the hypothesis that the prevention of nuclear accumulation of HDAC4 caused by PMS is NO-mediated and also that MEF2-D export from nuclei happens in a NO-dependent manner. However, L-arginine treatment of hindlimb-unloaded rats did not lead to MEF-2D nuclear content increase, so the mechanisms of PMS action on MEF-2D nuclear content should be more complex than just restoration of NO content in soleus muscles [27].

AMPK can phosphorylate HDAC4 and export it from nuclei [68] and AMPK activity may be also controlled by NO [37,38]. In the present study, any effect of AMPK on HDAC4 nuclear import is unlikely since we have not observed any changes of AMPK phosphorylation in the unloading group. HDAC4 can be phosphorylated by other kinases, such as PKD and CaMKII. Probably the phosphorylation of HDAC4 and its export from the nucleus are due to other kinases.

The activity of p300 HAT can be controlled by ERK 1/2-mediated phosphorylation of p300, which enhances myosin heavy chain I/beta gene expression via NFATc1 acetylation [69]. ERK1/2 is, in turn, activated by Y204 phosphorylation. The decrease in Y204 phosphorylated nuclear ERK2 was shown earlier after hindlimb unloading [67]. Plantar mechanical stimulation prevented ERK1/2 Y204 phosphorylation decrease, and this effect was blocked in the NOS-inhibited group. This result is in an agreement with the finding that the NOS inhibitor (L-NAME) treatment reduced nuclear ERK2 phosphorylation level in the tumor tissues of xenograft mice [70]. However, ERK2 total nuclear content decreased in all unloaded groups (Figure 7C). This suggests that the activity of the nuclear ERK2 is unlikely to be regulated by its traffic to the nucleus. Therefore, NO-dependent phosphorylation of ERK1/2 may also contribute to the PMS effects by activating p300-dependent mechanisms of slow myosin mRNA transcription regulation.

**Figure 8:**
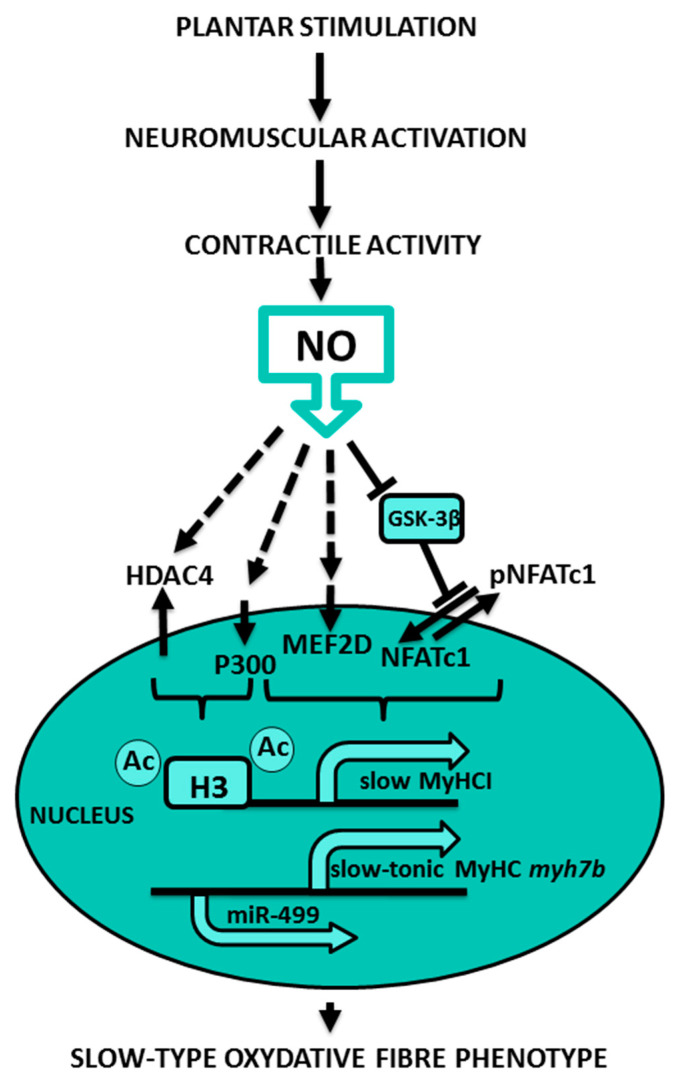
NO-dependent mechanisms of skeletal muscle fiber-type regulation during PMS.

The summary of observed NO-dependent mechanisms of skeletal muscle fiber-type regulation during PMS is shown in Figure 8.

Summing up, we can conclude that NO-synthase is an essential regulator of the unloading-induced fiber-type shift in soleus muscle, and the effects of plantar mechanical stimulation on slow-to-fast fiber-type ratio are mediated by prevention of nitric oxide decrease in the muscle tissue. The prevention of the unloading-induced NO content decrease by PMS prevents inactivation of micro-RNA-dependent signaling, dysregulation of nuclear contents of slow myosin transcription activators and repressors, and a decline of histones acetylation.

## 4. Materials and Methods

### 4.1. Ethical Approval 

All animal procedures were reviewed and approved by the Biomedicine Ethics Committee of the Institute of Biomedical Problems of the Russian Academy of Sciences/Physiology Section of the Russian Bioethics Committee (protocol no. 414, 23.12.2015). All experiments were performed in strict accordance with the American Physiological Society’s Guiding Principles in the Care and Use of Vertebrate Animals in Research and Training. Animals were housed in a temperature-controlled room on a 12:12 h light–dark cycle with food pellets and water provided ad libitum. Wistar male rats were obtained from the certified nursery for laboratory animals of the Institute of Bioorganic Chemistry of the Russian Academy of Sciences (Pushchino, Moscow region). On completion of the experiments, the animals were euthanized by intraperitoneal injection of a tribromoethanol overdose (750 mg/kg) followed by cervical dislocation.

### 4.2. Study Design and L-NAME Treatment

Three-month-old male Wistar rats weighing 180–225 g were randomly assigned to four groups (8 animals in each): vivarium control group, hindlimb suspended group for 7 day (7HS), hindlimb suspended group for 7 days subjected to daily PMS (7HS + PMS), hindlimb suspended group for 7 days subjected to daily PMS with the introduction of a nitric oxide synthase inhibitor (L-NAME) at a concentration of 90 mg/kg body weight per day intraperitoneally (7HS + LN + PMS). All other experimental groups received a placebo equivalent in volume. The experimental design did not include rats that received L-NAME only, or a group that received 7 days of hindlimb unloading and L-NAME. L-NAME reduces NO production, which results in increased total peripheral resistance and high blood pressure [71,72], so profound NO deficiency caused by L-NAME during HS leads to a high probability of stroke, atherosclerosis, myocardial infarction and congestive heart failure. We also suggest that the effect of L-NAME on unloaded and non-unloaded animals would not be equal, as the unloading-induced changes include not only an NO decrease, but calcium, myokines, ATP/ADP ratio, etc., so the consequences of NO modulation under these signaling conditions may vary from NO modulation in non-unloaded soleus, so we did not include a C + L-NAME group.

After the experiment, the rats were euthanized as described above, and their soleus muscles were dissected and immediately frozen in liquid nitrogen. The animals from the vivarium control groups were euthanized on the same day as the 7HS, 7HS + PMS and 7HS + LN + PMS groups. Both soleus muscles of each animal were collected. For the biochemical and immunohistochemical analysis we took the material of right soleus muscle. 

### 4.3. Hindlimb Suspension

Gravitational unloading was simulated by using a standard hindlimb suspension (7HS) model (Morey-Holton ER, Globus RK.1985). A strip of adhesive tape was applied to the animal’s tail, which was suspended by passing the tape through a swivel that was attached to a metal bar on the top of the cage. This allowed the forelimbs to have contact with the grid floor and allowed the animals to move around the cage for free access to food and water. The suspension height was adjusted to prevent the hindlimbs from touching any supporting surface while maintaining a suspension angle of ~30°. This model causes atrophy of the postural muscles, and subsequent recovery of the hindlimbs evokes muscle regeneration, resulting in the restoration of muscle mass and functioning.

### 4.4. Plantar Mechanical Stimulation

**Figure 9:**
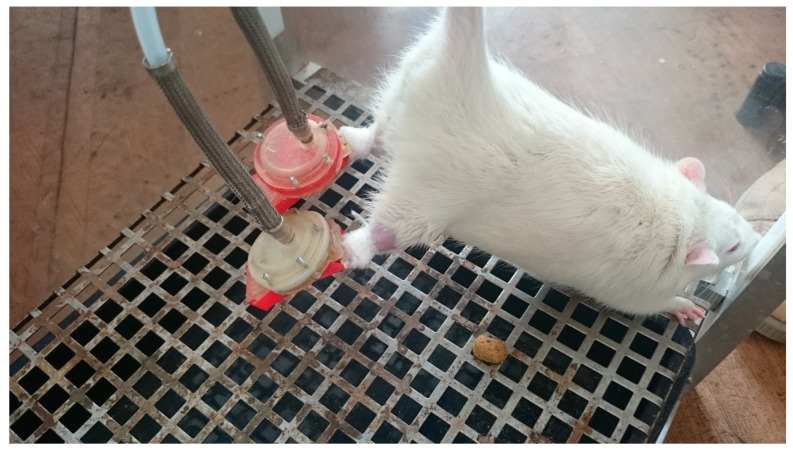
A photo of PMS application.

PMS was provided by a model previously described by Kyparos et al. (Kyparos et al. 1985), with modifications. The PMS apparatus is a plastic custom-built boot with a movable platform inside that allows regulation of pressure and frequency of sole stimulation. The PMS apparatus was attached to the animal’s foot above the ankle with an adhesive patch. Pressure was applied to the foot by the movable platform contacting the sole of the foot with an electronically controlled custom-built air pump attached to a hose. A photo of PMS application is shown in Figure 9.

Each sole was stimulated with a frequency of 1-s inflation, 1-s deflation for a total of 20 min, followed by 10 min of rest. The apparatus was removed after all cycle completions. This cycle was repeated eight times over a 4 h period during 7 days of unloading for the 7HS + PMS group and was performed during each day of the 7 day HS period for the 7HS + LN + PMS group. Pump cycling time and duration were controlled by a microprocessor. To sufficiently stimulate cutaneous mechanosensory receptors within the sole of the animal foot, pressure that exceeds their mechanical threshold (i.e., >8 mN) must be applied (Leem JW, Willis WD, Chung JM.1993). In Kyparos et al. (2005) the pressure required to stimulate the entire plantar surface was calculated as 13.9 mN/mm2 (Kyparos et al. 1985). This pressure was set in the present experiments.

### 4.5. NO Content Detection

NO content in soleus muscles was analyzed as described previously [34]. Briefly, relative intramuscular nitric oxide (NO) content was determined using a standard spin trap and electron paramagnetic resonance technique (EPR). We used diethyldithiocarbamate (DETC) as a spin trap that forms in tissues nitrosyl paramagnetic complexes with iron, which is in equilibrium with stationary NO concentration within the tissue and have a characteristic EPR spectrum. The spin trap was injected into rat soleus muscles at the rate of 500 mg/kg body mass. Immediately after the injection of DETC, aqueous solution of 29 mM FeSO4 mixture with 116 mM sodium citrate (2 mL/kg body mass) was injected in rat soleus muscle. After 30 min, the animals were sacrificed as described above and their soleus muscles were frozen in liquid nitrogen. The EPR signal was registered on a Bruker EMX-8 EPR spectrometer.

### 4.6. Nuclear and Cytoplasmic Extracts Preparation

Nuclear extracts were prepared from 50 mg of soleus muscle using NE-PER Nuclear and Cytoplasmic Extraction Reagents (Thermo Scientific, Waltham, MA, USA). Complete Protease Inhibitor Cocktail (Santa-Cruz), Phosphatase Inhibitor Cocktail B (Santa Cruz), PMSF (1 mM), aprotinin (10 μg/mL), leupeptin (10 μg/mL), and pepstatin A (10 μg/mL) were used to maintain extract integrity and function. Nuclear extracts were dialyzed by means of Amicon Ultra-0.5 centrifuge filters (Millipore, Burlington, MA, USA).

The protein content of all samples was quantified twice using a Quick Start Bradford Protein Assay (Bio-Rad Laboratories) in order to calculate the optimal sample value for electrophoretic gel. The supernatant fluid was diluted with 2× sample buffer (5.4 mM Tris-HCl, pH 6.8, 4% SDS, 20% glycerol, 10% β-mercaptoethanol, 0.02% bromphenol blue) and stored at −85 °C for immunoblot procedures.

### 4.7. Total Protein Fraction Preparation

Total muscle protein fractions were prepared from 10 mg of soleus muscle tissue cryosections, homogenized for 25 min in 100 μL RIPA Lysis Buffer System (Santa Cruz, CA, USA) with 10 mM EDTA, 50 mMβ-glycerophosphate, 0.5 mM DTT, 10μg/mL aprotinin, 10μg/mL leupeptin, 1 mM PMSF, 50 mM NaF, 1 mM Na3VO4, 10μg/mL pepstatin, and 20μL “complete Mini Protease Inhibitor Cocktail.” After that the samples were centrifuged at 20,000× *g* for 25 min. The protein content of all samples was quantified twice, diluted in 2× sample buffer and stored at −85 °C 

### 4.8. Immunoblots

For each protein analyzed, conditions of electrophoresis and Western blotting were optimized, according to the protein molecular weight and quantity in the lysate. Electrophoresis was carried out in the 10% separating polyacrylamide gel (0.2% methylene-bisacrylamide, 0.1% SDS, 375 mM Tris-HCl, pH 8.8, 0.05% ammonium persulfate, 0.1% TEMED) and in the 5% concentrating polyacrylamide gel (0.2% methylene-bisacrylamide, 0.1% SDS, 125 mM Tris-HCl, pH 6.8, 0.05% ammonium persulfate, 0.1% TEMED). The cathode (192 mM Tris-glycine, pH 8.6, 0.1% SDS) and anode (25 mM Tris-HCl, pH 8.6) buffers were used. Samples were loaded at the rate of 25 μg of total protein in each sample. The samples of each group were loaded on the gel together with control samples. Electrophoresis was carried out at 17 mA/gel in a mini system (Bio-Rad Laboratories) at room temperature.

Electrotransfer of the proteins was carried out in buffer (25 mM Tris, pH 8.3, 192 mM glycine, 20% ethanol, 0.04% SDS) onto nitrocellulose membrane at 100 V and 4 °C in the mini Trans-Blot system (Bio-Rad) for 120 min. The membranes were blocked in 5% non-fat dry milk solution (Bio-Rad) in PBST (phosphate-buffered saline pH 7.4, 0.1% Tween 20) for 1 h at room temperature. To reveal protein bands, the following primary polyclonal antibodies were used: total GSK-3β (1:1000, Cell Signaling Technology, Danvers, MA, USA, # 12456), phosphorylated Ser 9 GSK-3β (1:1000, Cell Signaling Technology, USA, # 9322), GAPDH (1:, Applied Biological Materials Inc., Richmond, BC, Canada, # G041), Lamin B1 (1:500, Abcam, Cambridge, MA, USA, # ab16048), MEF2-D (1:1000, EMD Millipore, USA, # AB2263), acetyl-Histone H3 (1:1000, EMD Millipore, USA, # 06-599), total Histone H3 (1:1000, Cell Signaling Technology, USA, # 9715), HDAC4 (1:500, Cell Signaling, USA, #2072), HAT P300 (1:500, Abcam, USA, # ab231010), phosphorylated Y204 ERK2 (1:500, Cell Signaling Technology, USA, # 9101), total ERK2 (1:2000, Cell Signaling Technology, USA, # 4695), NFATc1 (1:1000, Abcam, USA, # ab2796), phosphorylated Thr 183/172 AMPKα1/2 (1:1000, ABM, San Francisco, CA, USA, # Y408289), AMPKα (1:1000, Cell Signaling Technology, USA, #2532), phosphorylated Tyr 180/Thr182 MAP kinase p38 (1:500, GeneTex, Inc., Irvine, CA, USA, # GTX59567), total MAP kinase p38 (1:500, Cell Signaling Technology, USA, #9212), total neuronal NO-synthase (1:500, BD Transduction, San Jose, CA, USA, #610309). All the primary antibodies were used for overnight incubation at 4 °C. The secondary HRP-conjugated antibodies (goat-anti-rabbit, Santa Cruz, 1: 30,000, goat-anti-mouse, Santa Cruz, 1: 25,000) were used for a 1 h incubation at room temperature. The blots were washed 3 times, 10 min each, in PBST. Then the blots were revealed using the ImmunStar TM Substrate Kit (Bio-Rad Laboratories, USA) and the C-DiGit Blot Scanner (LI-COR Biotechnology, Lincoln, NE, USA). Protein bands were analyzed using the Image Studio Digits Ver. 4.0 software. All image densities were measured in linear range of scanner detection. The optical absorption (OA) of the control group band on analytical membrane was taken as 100%, while the OA of other groups was compared with that of the control group bands localized on the same membrane. The blots on which phosphorylated proteins were detected were stripped with RestoreWestern Blot Stripping Buffer (Thermo Scientific) and then re-probed with total protein antibodies overnight at 4 °C to analyze the phosphorylation level of the proteins. Then the blots were incubated with HRP-conjugated goat-anti-rabbit secondary antibody and visualized as described above. It was controlled that phosphorylated proteins—goat-anti-rabbit-HRP complexes were washed out completely from the blots. The blots were washed 3 × 10 min at room temperature with PBST after incubations with antibodies and Restore Western Blot Stripping Buffer. Nuclear fraction protein signals were normalized to lamin B1; phosphorylated proteins were normalized to total protein content. Ponceau S staining of the membranes was utilized to ensure equal loading of the extracts (not shown). All Western blots were repeated at least three times.

### 4.9. RNA Isolation

Total RNA was extracted from frozen soleus muscle samples using RNeasy Micro Kit (Qiagen, Germany) according to the manufacturer’s protocol. RNA concentration was analyzed at 260 nm. RNA quality of purification was evaluated according to 260/280 and 260/230 ratios, and its integrity was assessed by gel electrophoresis with ethidium bromide staining of 1 μg total RNA on 1% agarose gel. The RNA solutions were stored frozen at −85 °C and were used in RT-PCR procedures.

### 4.10. RT-qPCR

Reverse transcription was performed by incubation of 0.5 μg of RNA, random hexamers d(N)6, dNTPs, RNase inhibitor and MMLV reverse transcriptase for 60 min at 42 °C. The samples to be compared were run under similar conditions (template amounts, duration of PCR cycles). The annealing temperature was based on the PCR primers’ optimal annealing temperature. PCR primers used for RNA analysis are shown in Table 2. The amplification was real-time monitored using SYBR Green I dye and the iQ5 Multicolor Real-Time PCR Detection System (Bio-Rad Laboratories, USA). To confirm the amplification specificity, the PCR products from each primer pair were subjected to a melting curve analysis, and sequencing of the products was provided at least once. Relative quantification was performed based on the threshold cycle (CT value) for each of the PCR samples [73]. *RPL19* was tested and chosen for the normalization of all quantitative PCR analysis experiments in the current study.

### 4.11. miRNA Isolation

Total RNA, including miRNA, was purified using the miRNeasy Micro Kit (Qiagen, Germany) from frozen soleus muscle samples according to the manufacturer’s protocol. Isolated total RNA including miRNA was used as a template for reverse transcription followed by real-time PCR. MiScript II RT Kit (Qiagen, Hilden, Germany) was used to perform a reverse transcription. The reverse transcription was performed by incubation of the mix of template RNA, miScript Reverse Transcriptase Mix, 10× miScript Nucleics Mix and 5× miScript HiFlex Buffer for 60 min at 37 °C and then for 5 min at 95 °C according to the manufacturer’s protocol. Reverse transcription was performed in a thermocycler (CFX96 Touch Real-Time PCR Detection System, Bio-Rad Laboratories, Hercules, CA, USA). The cDNA was used as a material for real-time PCR.

### 4.12. miRNA RT-qPCR

For real-time PCR we used miRNA-499 and miRNA-208 miScript Primer Assays and the miScript SYBR Green PCR Kit, which contains the miScript Universal Primer (reverse primer) and QuantiTect SYBR Green PCR Master Mix (Qiagen, Germany).

PCR was performed by initial activation step for 15 min at 95 °C, and 3-step cycling, including denaturation step for 15 s at 94 °C, annealing step for 30 s at 55 °C and extension step for 30 s at 70 °C. The total number of cycles was 40.

To analyze the cDNA content of various microRNAs in each sample, we used the values of Ct (test) and Ct (ref), where Ct (ref) is the intersection point of the baseline and the amplification graph of the reference gene (RNU-6) in the sample, and Ct (test) is the intersection point of the baseline and a graph of amplification of the studied gene in the same sample.

### 4.13. MyHC Immunostaining

The transverse frozen sections (10 μm thick) of the soleus muscle samples were prepared with a Leica CM 1900 cryostat (Leica, Braunschweig, Germany) at −20 °C, dried at room temperature for 15 min, and incubated in PBST for 20 min. Sections were incubated with primary antibodies MyHC I(β) slow, 1:100 (Sigma, St. Louis, MO); MyHC fast, 1:60 (DSMZ) for 1 h at +37 °C. The anti-MyHC fast antibody used in this study does not distinguish between different fast MyHC isoforms. After three 10-min washes with PBST, the sections were incubated with secondary antibodies (Alexa Fluor 546 (1:1000) and Alexa Fluor 350 (1:1000); Molecular Probes, Waltham, MA, USA) for 60 min in the dark at room temperature. Subsequently, the sections were washed three times with PBST, examined, and photographed with a Leica Q500MC fluorescent microscope at magnification ×400. For myofiber immunohistochemical evaluation of the slow-to-fast fiber-type ratio the scanned digitized images of immunostained cryosections were processed by ImageJ software. On the basis of outer membrane borders, we determined the percentage of slow-type and fast-type fibers of given oval/circle planes that matched with the cross-sectioned myofiber profiles of slow MyHC positive and fast MyHC positive (fast MyHC and slow MyHC positive). The percentage of different muscle fiber types was evaluated relative to all muscle fibers present in each section. At least 10 cross sections per sample were analyzed to determine the percentage of different muscle fiber types in the sample (*n* = 8).

## 5. Statistical Analysis

All data are expressed as median and interquartile range (0.25–0.75) of eight animals. The means of all groups are shown as a percentage of the control group. To check whether the differences among groups were statistically significant, given the small sample sizes and comparisons between four groups, we adopted the Kruskal–Wallis nonparametric test, which is statistically informative despite the small number of subjects in each group, followed by Dunn’s post hoc test. A *p*-value < 0.05 was regarded as statistically significant.

## Figures and Tables

**Table 1:** Relative NO content in rat soleus muscle in the control group (C), 7-day hindlimb-suspended group (7HS), 7-day hindlimb suspended group with plantar mechanical stimulation (7HS + PMS), 7-day hindlimb suspended group with plantar mechanical stimulation+ L-NAME introduction (7HS + LN + PMS). *—significant difference from control group (*p* < 0.05).

Group	*n*	EPR Signal (M ± m), Relative Units
**C**	8	1.00 ± 0.01
**7HS**	8	0.41 ± 0.07 *
**7HS + PMS**	6	0.83 ± 0.05
**7HS + LN + PMS**	7	0.42 ± 0.08 *

**Table 2:** Primers used for RT-PCR analysis.

Gene Description	Forward Primer Reverse Primer	GenBank
***Myh7 (MyHC I(β))***	5′-ACAGAGGAAGACAGGAAGAACCTAC-3′ 5′-GGGCTTCACAGGCATCCTTAG-3′	NM_017240.2
***Myh7b***	5′-GAGTGTGGAGCAGGTGGTATTT-3′ 5′-GGACCCCAATGAAGAACTGA-3′	NM_001107794.2
***SOX6***	5′-TCAAAGGCGATTTACCAGTGAC-3′ 5′-TTGTTGTGCATTATGGGGTGC-3′	NM_001024751.1
***Rcan1 (MCIP1.4)***	5′-CCGTTGGCTGGAAACAAG-3′ 5′-GGTCACTCTCACACACGTGG-3′	NM_153724.2
***RPL19***	5’- GTACCCTTCCTCTTCCCTATGC-3’ 5’- CAATGCCAACTCTCGTCAACAG-3’	NM_031103.1
***Myh2 (MyHC IIa)***	5′-TATCCTCAGGCTTCAAGATTTG-3′ 5′-TAAATAGAATCACATGGGGACA-3′	NM_001135157.1
***Myh4 (MyHC IIb)***	5′-CTGAGGAACAATCCAACGTC-3′ 5′-TTGTGTGATTTCTTCTGTCACCT-3′	NM_019325.1
***Myh1 (MyHC IId/x)***	5′-CGCGAGGTTCACACCAAA-3′ 5′-TCCCAAAGTCGTAAGTACAAAATGG-3′	NM_001135158.1
***Myoz1 (Calsarcin-2)***	5′-GTGGAACTTGGCATTGACCT-3′ 5′-GAGGACCAAGGGTTCACTCA-3′	NM_001109097.1
***Rcan1 (MCIP 1.4)***	5′-CCGTTGGCTGGAAACAAG-3′ 5′-GGTCACTCTCACACACGTGG-3′	NM_153724.2
***NOS1***	5′-GACAACGTTCCTGTGGTCCT-3′ 5′-TCCAGTGTGCTCTTCAGGTG-3′	NM_052799.2

## Data Availability

Data available in a publicly accessible repository.
